# Preparation and characterization of tannin-composite soy protein-based bio-adhesives

**DOI:** 10.1039/d6ra04507c

**Published:** 2026-08-17

**Authors:** Xun Zhou, Yunfeng Tao, Hualong Xuan, Yixi Zhang, Huali Tang

**Affiliations:** a College of Biology and Food Engineering, Chongqing Sanxia University of Science and Technology Chongqing 404100 China hualidfood@163.com; b College of Materials and Chemical Engineering, Southwest Forestry University Kunming 650224 China

## Abstract

Developing sustainable wood adhesives from renewable biomass is essential for reducing dependence on petroleum-derived adhesives and promoting environmentally friendly wood manufacturing. In this study, an environmentally friendly tannin composite soy protein adhesive was successfully prepared by adding tannin-based resin (TR) and silica (SiO_2_) to improve its water resistance and wet shear strength. The structure, curing characteristics, thermal properties, and adhesion performance of the obtained soy protein adhesives were determined by Fourier transform infrared spectroscopy (FTIR), differential scanning calorimetry (DSC), thermogravimetric analysis (TGA) and wet shear strength. When TR was 10%, the soy protein adhesive without adding SiO_2_ had a wet shear strength of 0.84 MPa at 63 °C. After further adding SiO_2_, the adhesive achieved a wet shear strength of 1.59 MPa at 93 °C, attributed to its densely cross-linked network comprising Schiff base, inorganic-organic hybrid interactions, and non-covalent hydrogen bonding. This work not only overcomes the water resistance issue of traditional soy protein adhesives, but also provides a feasible method for the development of biomass adhesives. It has the potential to replace petroleum-based products and is particularly suitable for the field of wood composite materials with high weather resistance requirements.

## Abstract 2

A bio-based adhesive composed of tannin resin (TR), soy protein isolate (SPI), and nano-SiO__2__ is developed, where covalent cross-linking (TR-SPI) and hydrogen bonding (SiO__2__-SPI) synergistically reinforce interfacial adhesion, enabling the fabrication of high-performance plywood *via* heat pressing.

## Graphical abstract

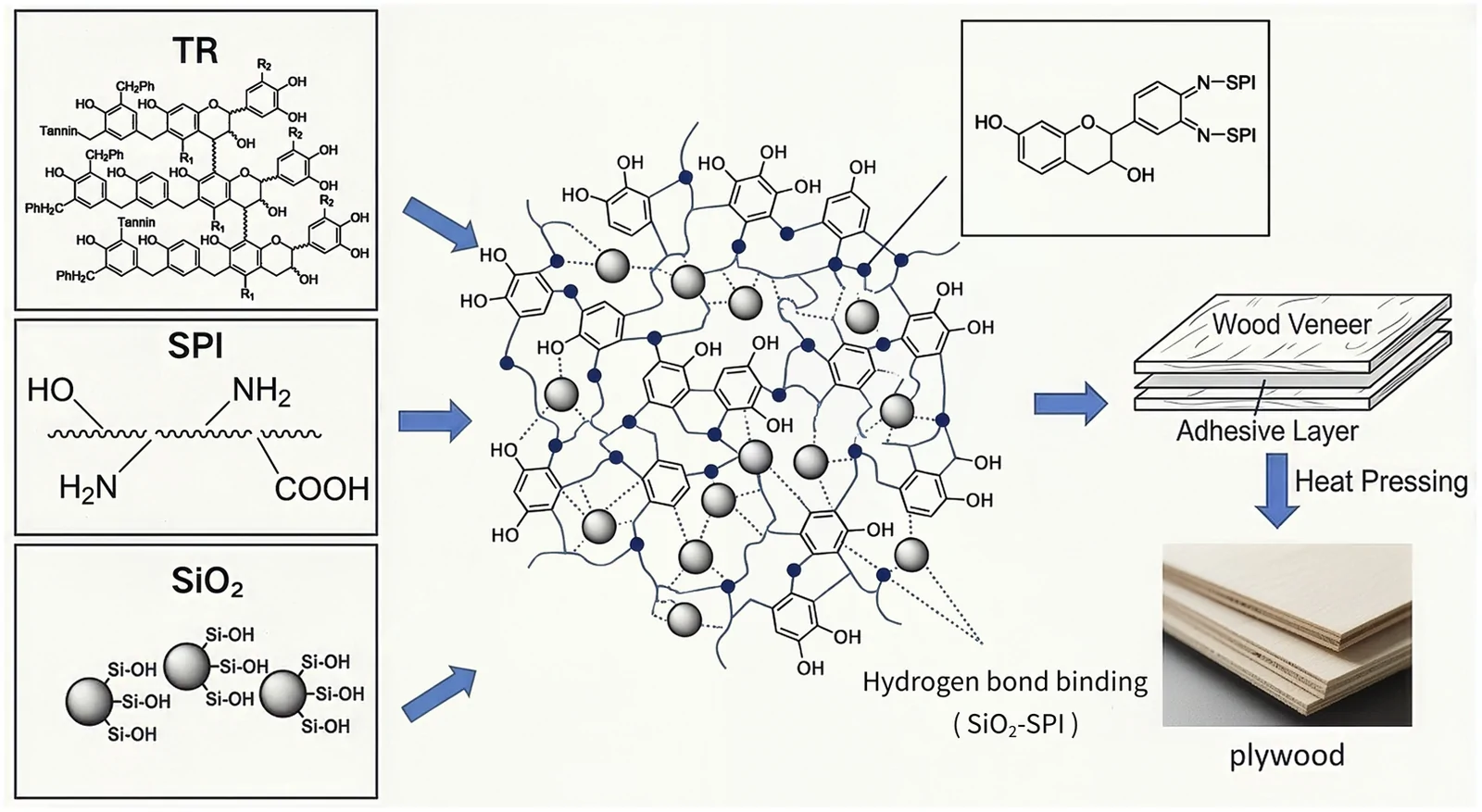

## 1. Introduction

Traditional aldehyde resin adhesives are widely used in the wood processing industry, but the formaldehyde they release is harmful to the environment and human health, so there is an urgent need for formaldehyde-free alternatives.^1^ Meanwhile, with the global demand for sustainable development and circular economy growing, promoting the resource utilization of agricultural and forestry by-products and developing bio-based materials have become important ways to address resource and environmental challenges.^2,3^ Natural products such as plant protein, cellulose, lignin, tannin and starch are renewable and environmentally friendly, making them excellent raw materials for preparing wood adhesives^4,5^ Soy protein is regarded as one of the ideal raw materials for the development of bio-based materials due to its abundant sources and easy availability.^6^ However, its molecular structure contains a large number of hydrophilic groups, resulting in poor water resistance and limited shear strength in the adhesive,^7^ which requires modification treatment.

At present, the modification methods for improving the performance of soy protein adhesives mainly include crosslinking modification, acid-base treatment, surfactant treatment and grafting modification.^8^ Crosslinking modification involves introducing reactive chemical substances to react with hydroxyl (–OH), amino (–NH_2_), and carboxyl (–COOH) groups in soy protein molecules, thereby constructing a dense crosslinking network structure within the protein and enhancing the strength and water resistance of the adhesive.^9^ Epoxy compounds, isocyanate compounds, and various resin polymers are commonly used crosslinking agents.^10^ Hydrophilic groups are grafted onto bisphenol A E44 and an oil-in-water emulsion is prepared *via* a phase transfer process, in which the epoxy groups can form multiple stable cross-linked networks with soy protein molecules to improve the water resistance and thermal stability of the adhesive.^11^ Li *et al.* synthesized a thermally driven cationic polyurethane (CWPOU) crosslinker containing oxime-based isocyanate groups and catechol groups, and the modified soy meal adhesive exhibited excellent bonding performance, cold-pressing performance and antifungal performance.^12^ Yan *et al.* synthesized hyperbranched sulfuretted polyamidoamine-epichlorohydrin (PCDME) resin using citric acid, diethylenetriamine, and monothioglycerol, which formed multiple physical and chemical cross-linking networks with soy protein molecules, thereby enhancing the cohesive strength, high moisture resistance, and thermal stability of the adhesive.^13^ Although such crosslinking agents can effectively enhance the adhesive properties of soy protein, their synthetic raw materials derived from fossil fuels still pose challenges such as high production and preparation costs.^14^ Utilizing biomass resources for adhesives can reduce dependence on fossil fuels and better align with green and environmentally friendly principles.

Tannin is a natural polyphenolic compound that is widely present in plants, abundant in reserves and renewable.^15,16^ Moreover, due to its excellent antibacterial and antioxidant properties,^17,18^ it has been applied in the development of various functional materials.^19–23^ Condensed tannin is a condensate with flavane-3-alcohol as the basic structural unit. Its molecular structure contains a large number of highly reactive phenolic hydroxyl groups, which can partially replace phenol from petroleum sources in resin synthesis and is an ideal substitute for the preparation of adhesives.^24^ Ma *et al.* developed formaldehyde-free tannin-derived non-covalent (TNC) adhesives through multiple hydrogen bond interactions between tannin and polyvinyl alcohol (PVA), enabling high-performance noncovalent bonding to diverse substrates including wood, glass, and iron. They further derived a series of environmentally friendly, formaldehyde-free tannin adhesives sourced from larch and black wattle.^25^ Wang *et al.* used hydroxylated graphene (G-OH) as a green nucleophilic reagent to depolymerize acacia tannin, thereby preparing high-performance bio-based phenolic resins. This approach achieved efficient depolymerization of tannin, with the resulting product capable of replacing 50% of phenol in resin synthesis.^26^ Zhao *et al.* synthesized tannin-formaldehyde resin using tannin and formaldehyde. By replacing 10% of the formaldehyde with furfural in the reaction, they produced tannin-formaldehyde-furfural resin (TFFu), which reduced free formaldehyde content and enhanced thermal stability.^27^

In this study, tannins undergo phenolic condensation reactions, leading to molecular cross-linking and structural reorganization, thereby forming a tannin-based resin (TR) polymer characterized by a robust three-dimensional network architecture. Subsequently, the synergistic modification of soy protein isolate (SPI) with TA and silica (SiO_2_) markedly enhances both the water resistance and adhesive strength of SPI-based adhesives. Differential scanning calorimetry (DSC) was employed to investigate the curing characteristics of the adhesives, while thermogravimetric analysis assessed their thermal stability. Fourier transform infrared spectroscopy (FT-IR) was utilized to explore structural changes at the molecular level. Parameters such as reaction temperature and reactant ratios were optimized to prepare tannin-modified soy protein adhesives with superior performance. In comparison to conventional phenolic resins, the formulation reduces the quantity of phenol utilized, lowers raw material costs, and demonstrates potential for industrial application.

## 2. Materials and methods

### 2.1. Materials

Soy protein isolate (SPI, protein content ≥95%), formaldehyde solution (37%), sodium hydroxide, and phenol were purchased from Shanghai McLean Biochemical Technology Co., Ltd. Mimosa tannin (TA), industrial grade. Silicon dioxide was supplied by Tianjin Zhiyuan Chemical Reagent Co., Ltd. Poplar veneer dimensions were 20 cm × 12.5 cm × 2 mm with a moisture content of 10–12%, provided by Linyi City, Shandong Province, China.

### 2.2. Synthesis of tannin-based resin

Add 20 g of mimosa tannin, 80 g of molten phenol, 64 g of sodium hydroxide solution (40%) and 70 g of distilled water into a flask equipped with a condenser tube and a magnetic stirring device in a water bath and heat to 60 °C, and continue stirring until it reaches 85 °C. Add 50% formaldehyde solution to the reaction bottle and react for 30 minutes. Then add 54 g of formaldehyde solution (37%) again, stir and react at the same temperature for 50 minutes, repeat twice. Finally, the product is rapidly cooled to obtain tannin-based resin (TR). After the preparation of the above tannin-based resin is completed, the bonding strength of the plywood is explored, and SiO_2_ is added to the tannin-based resin and reacted for 30 minutes to obtain SiO_2_–TR resin for later use.

### 2.3. Synthesis of soy protein isolate-based adhesives

As shown in Table 1, 10 g of soy protein isolate and 40 g of water were mixed at room temperature and stirred rapidly for 9 minutes until completely dispersed and a homogeneous system was formed to obtain SPI adhesive. Subsequently, 5%, 10% and 15% of TR components by weight were added respectively based on the weight of SPI adhesive, and after thorough stirring, tannin composite soy protein adhesive was obtained. The modified adhesives are marked as 5% TR, 10% TR and 15% TR. For the preparation of SiO_2_–TR–SPI adhesives, SiO_2_ is reacted with TR resin at 60 °C for 30 min, followed by formulation of resins at varying SiO_2_ : TR : SPI mass ratios according to the established TR–SPI synthesis protocol.

**Table 1:** Formulations of SPI adhesive modified with different TR addition amounts

Adhesive	SPI (g)	Water (g)	TR (g)	SiO_2_ (g)
5% TR–SPI	10	40	2.5	—
10% TR–SPI	10	40	5	—
15% TR–SPI	10	40	7.5	—
10% SiO_2_–TR–SPI	10	40	5	5

### 2.4. Preparation of plywood

The experiment utilized 20 cm × 12.5 cm × 2 mm poplar veneers to prepare plywood specimens. Adhesive was applied to both sides of the core board at a rate of 200 g m^−2^ per side. After adhesive application, veneer layers were stacked with grain directions perpendicular to each other. The stack was hot-pressed at 160 °C under 1.1 MPa pressure for 6 minutes. The successfully pressed plywood was then removed and left at room temperature for 24 hours before performance testing.

### 2.5. DSC analysis

DSC analysis (Netzsch 200F3, Germany): weigh 6–7 mg of SPI, 10% TR–SPI and 10% SiO_2_–TR–SPI samples respectively into aluminium crucibles. Record sample mass before sealing for DSC curve testing. The heating rate is 10 °C min^−1^ and the temperature range is 30–250 °C.

### 2.6. Mechanical properties testing of plywood

The prepared plywood samples were placed at room temperature for 24 h, and then cut into 100 × 25 mm^2^ plywood specimens with a bonding area of 25 × 25 mm^2^. The cut plywood specimens were placed in 63 ± 3 °C hot water and 93 ± 3 °C boiling water for heating for 3 hours. After being taken out and cooled to room temperature for 10 min, the adhesive strength were conducted. The dry and wet shear strengths of the plywood were tested in accordance with the relevant national standards GB/T 9846-2015. The adhesive strength was tested on a universal testing machine (ETM10B type, Shenzhen, China) at a test speed of 10 mm min^−1^. Each data in the bond strength test is the average of at least seven samples. The average value of shear strength was determined based on at least seven replicate experiments.

### 2.7. Fourier transform infrared (FTIR) spectroscopy

The dried samples were ground into powder by mixing with potassium bromide (KBr) at a mass ratio of 1 : 100. Testing was conducted using an infrared spectrometer (Nicolet IS50, USA). The wavenumber range was set from 500 to 4000 cm^−1^, with 32 scans performed at a resolution of 4 cm^−1^ to enhance data reliability and analytical accuracy.

### 2.8. Measurement of thermal properties

The thermal stability of the samples was analysed using a thermogravimetric analyser (TGA 55 Discovery 0550-1583). Prior to TG testing, the samples underwent freeze-drying. Approximately 10 mg of sample was weighed and placed in a crucible. Under a nitrogen atmosphere, the thermal stability was tested at a heating rate of 10 °C min^−1^ within the temperature range of 30–800 °C.

## 3. Results and discussion

### 3.1. Curing characteristics

**Fig. 1:**
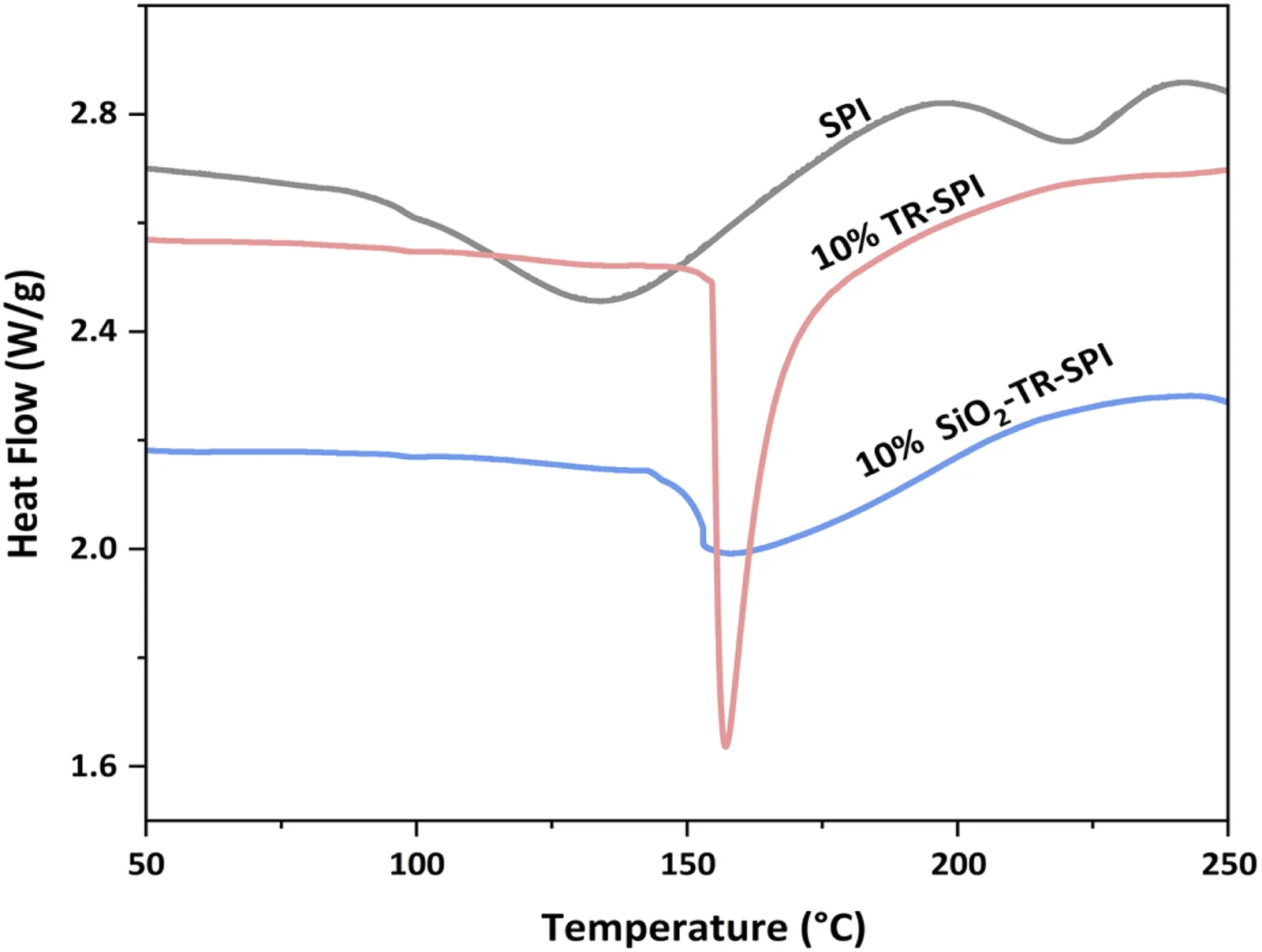
DSC curves of TR–SPI series adhesives.

Curing temperature is an important indicator for detecting thermosetting adhesives. To further study the curing properties of SPI, 10% TR–SPI and 10% SiO_2_–TR–SPI adhesives, DSC analysis was performed on three samples. As shown in Fig. 1, unmodified SPI shows curing behavior in a lower temperature range. Its curing starting temperature is around 100 °C and reaches a peak at 134 °C, indicating that the active groups contained in SPI itself can react under heating conditions, but the cross-linked network formed is weak and has limited thermal stability, which is consistent with the TG results. The curing starting temperature of 10% TR–SPI is at 154 °C and reaches a peak at 157 °C; the curing starting temperature of 10% SiO_2_–TR–SPI is around 148 °C and reaches a peak at 159 °C. Compared with the SPI adhesive, the curing temperature of the 10% SiO_2_–TR–SPI adhesive is lower and slightly higher than the 10% TR–SPI sample. The addition of SiO_2_ reduces the curing temperature and accelerates the curing of the adhesive at low temperatures. This may be because SiO_2_ serves as an additive to strengthen the interface bonding strength with the TR–SPI adhesive, so it has a lower curing temperature and a better degree of curing.

### 3.2. Shear strength analysis

#### 3.2.1 Effect of different tannin-based resin addition levels on the shear strength of TR–SPI

**Fig. 2:**
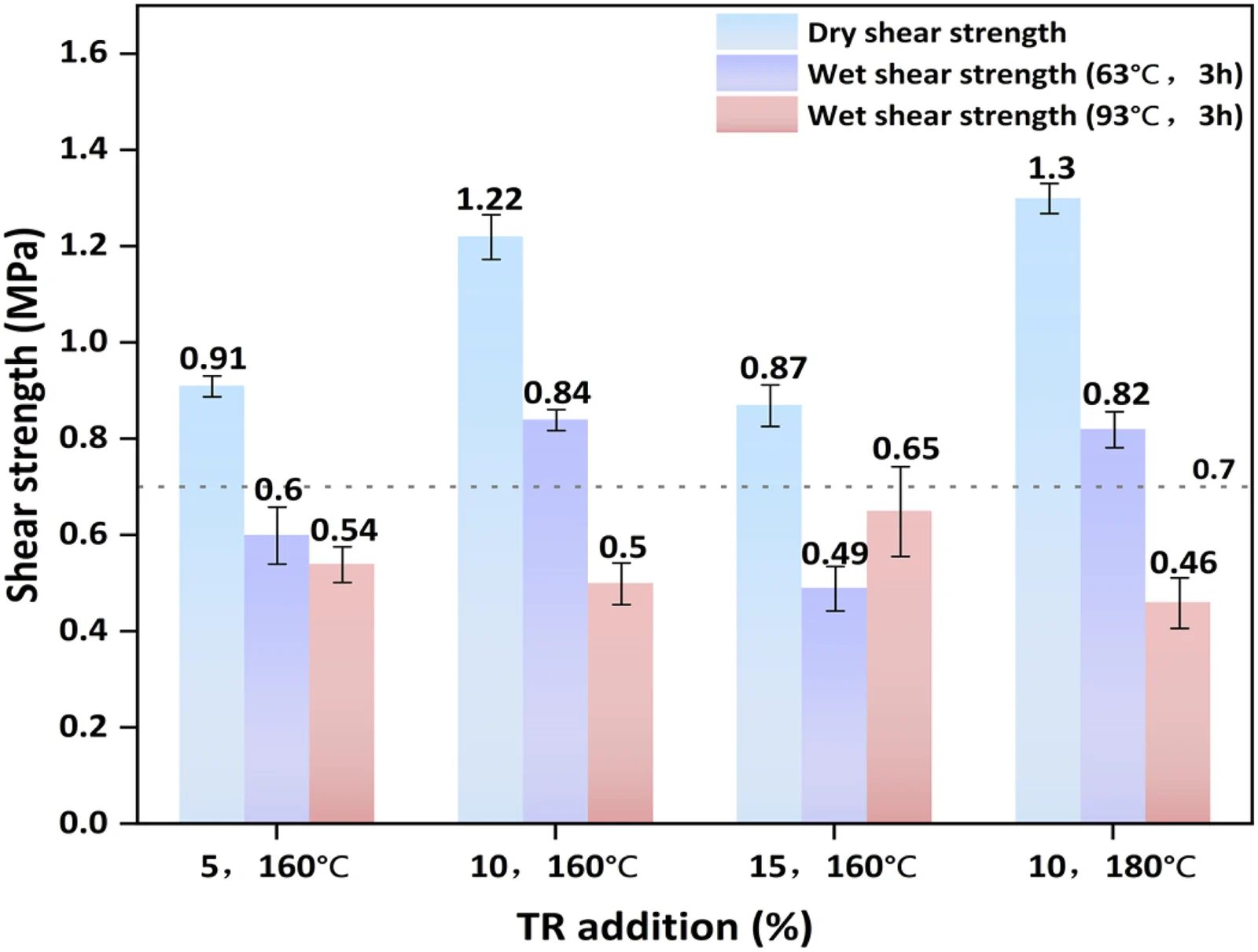
Shear strength of TR–SPI series adhesives.

Fig. 2 shows the shear strength of TR–SPI adhesives at hot-pressing temperatures of 160 °C and 180 °C. The dry strength of TR–SPI adhesives with 5%, 10%, and 15% TR addition all meets the requirements for Class II plywood in the Chinese national standard (≥0.7 MPa). As the proportion of TR increases from 5% to 10%, the shear performance also improves. This may be attributed to the Schiff base reaction between the hydroxyl groups of the TR resin and the active groups of SPI, forming a cross-linked network structure. However, when the amount of TR is 15%, the bonding strength decreases instead. On one hand, the increased content of unreacted TA in the TR resin weakens the water resistance. On the other hand, the excessive unreacted TR resin fails to form a cross-linked network, which also weakens the water resistance. To enhance the shear strength, on this basis, with 10% TR as the target, increasing the hot-pressing temperature from 160 to 180 °C leads to a slight increase in the bonding strength,^28^ but the impact on the bonding performance is not significant.

As shown in Fig. 2, the wet shear strength of plywood specimens was tested after immersion in 63 °C hot water for 3 hours. The shear strength improved significantly as the TR resin content increased from 5 to 10%. However, when the TR content reached 15%, the bonding strength decreased. This decline may be attributed to an increase in hydrophilic hydroxyl groups from tannins failing to bond with soy protein isolate, thereby weakening water resistance. Secondly, upon raising the hot-pressing temperature to 180 °C, the wet strength measured was 0.82 MPa. Comparative analysis revealed that temperature exerted negligible influence on bond strength. To further verify the bonding strength of the resin, three-layer plywood specimens were immersed in boiling water at 93 °C for 3 h to test their mechanical strength. When compared with the strength at 63 °C, it was found that the bonding strength of TR–SPI adhesives with 5%, 10% and 15% addition amounts all decreased. This indicates that high temperature impairs the bonding performance of the adhesives, and the water resistance of TR–SPI needs to be further improved. Although the plywood at 63 °C meets the national standard, the wet strength at 93 °C does not meet the standard yet.

#### 3.2.2 Research on the bonding performance of SiO_2_–TR–SPI adhesive

To address the water resistance of the adhesive, SiO_2_ was incorporated into the tannin-based resin at 5% by weight and reacted for 0.5 hours. Subsequently, SiO_2_–TR–SPI adhesives were prepared using SiO_2_–TR components at 5%, 10%, and 15% by weight. Dry strength and wet strength at 63 °C and 93 °C were evaluated.

**Fig. 3:**
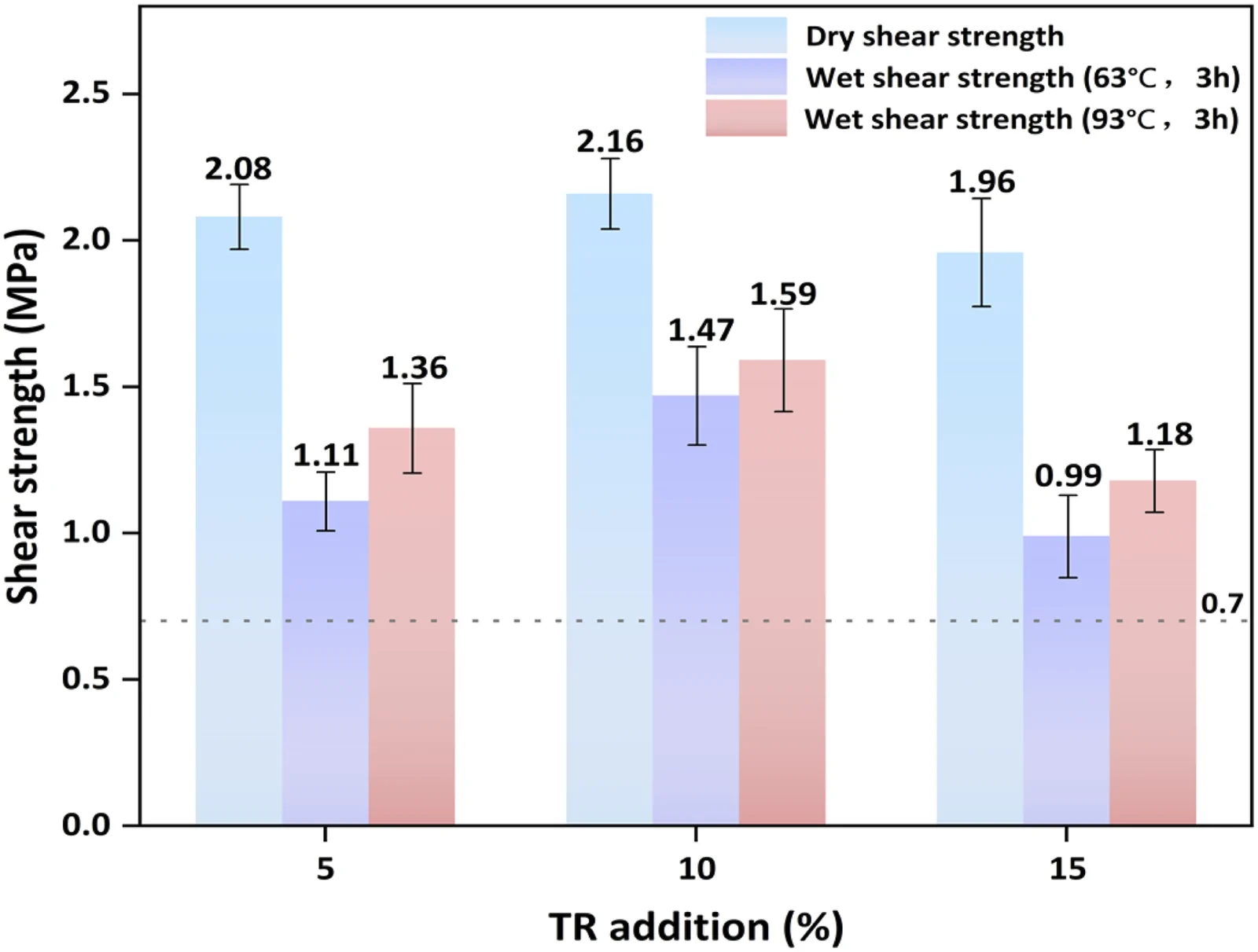
Shear strength of SiO_2_–TR–SPI series adhesives.

Nano-SiO_2_ is an inorganic rigid particle filler. Due to its own small size effect, it can also be used as a modified material for adhesives. When nano-SiO_2_ particles are used to modify adhesives, through their microsphere structure and inorganic rigidity, they can enhance the bonding strength, improve thermal stability and regulate viscosity of the adhesives. At present, SiO_2_ particles have been applied in modified epoxy resin adhesives and chitosan-derived adhesives.^29,30^ Based on the excellent properties of SiO_2_, 5% SiO_2_ was added to the TR resin to prepare the SiO_2_–TR–SPI adhesive. As can be seen from Fig. 3, the dry shear strength of the plywood has been significantly improved compared to Fig. 2. The strength also increases with the increase of TR addition amount, but decreases when it reaches 15%, which is consistent with the experimental results mentioned above.

As shown in Fig. 3, after the plywood was soaked in water at 63 °C for 3 hours, the wet shear strengths of 5%, 10%, and 15% SiO_2_–TR–SPI adhesives all far exceeded the national standard (≥0.7 MPa). The wet shear strength of SiO_2_–TR–SPI at 93 °C is actually higher than that at 63 °C. Therefore, the experiment speculates that the addition of SiO_2_, on the one hand, fully contacts and interacts with the TR–SPI resin. Through physical adsorption and chemical bonding, a more stable cross-linked network structure is formed, significantly improving the viscosity, strength and water resistance of the adhesive. On the other hand, the addition of SiO_2_, through physical filling and reinforcement, improves the fluidity of the adhesive, enhancing the uniformity of application during the gluing process. Moreover, it is easier to penetrate into the tiny gaps existing on the wood surface, enabling the adhesive to form a nail structure during the curing process and significantly improving the mechanical properties of the plywood. This adhesive exhibits good water resistance, which means it has a longer service life and reduces resource consumption and waste generation, which is also a manifestation of its environmental friendliness.

### 3.3. Characterization of the structure of TR and different modified SPI adhesives

**Fig. 4:**
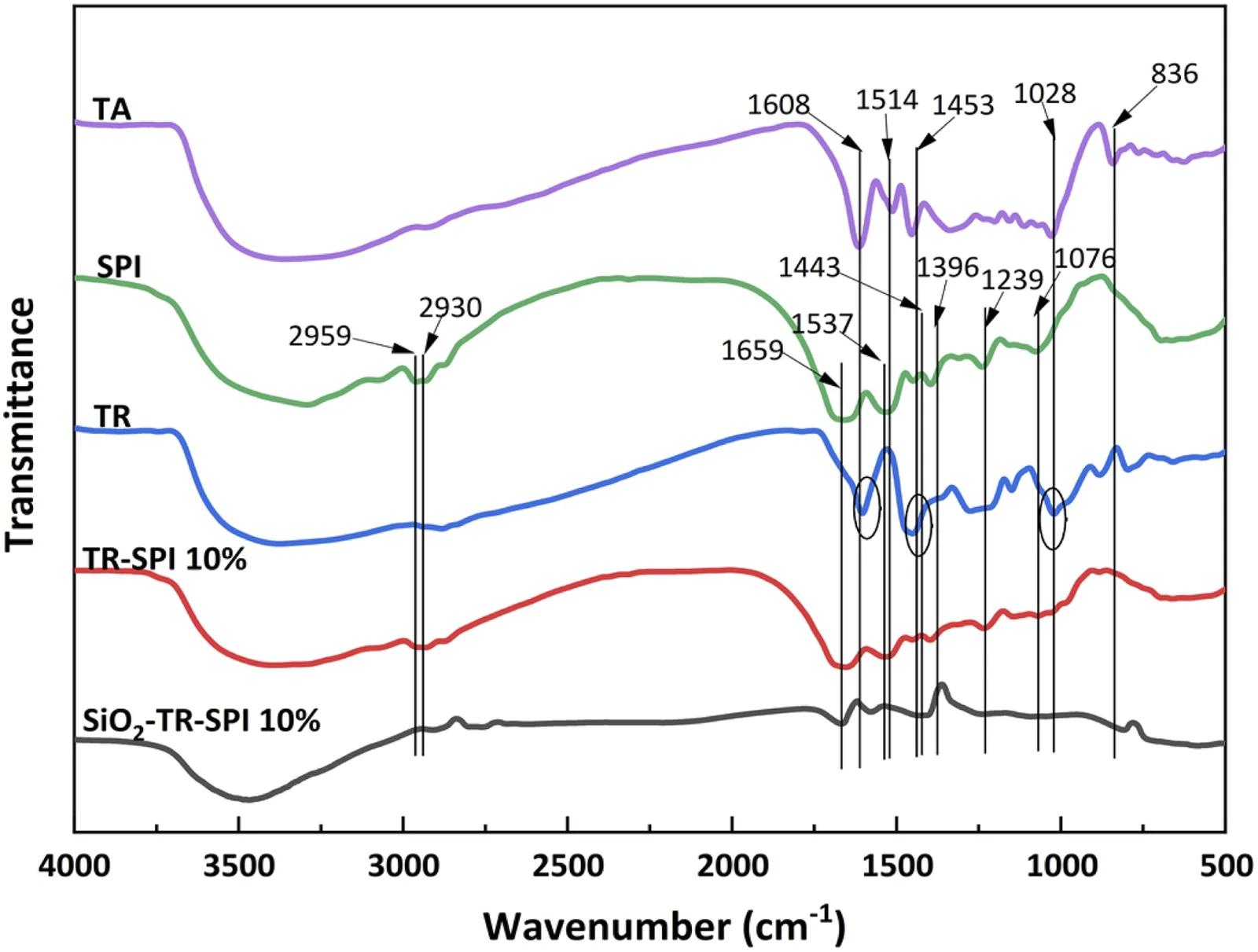
Infrared spectrum of SPI adhesive.

The FTIR test results of the SPI series adhesives are shown in Fig. 4. The broad peak of the TA curve near 3300 cm^−1^ originates from the stretching vibration of the phenolic hydroxyl group (–OH), and the sharp absorption peaks at 1608 cm^−1^ and 1514 cm^−1^ are the vibration characteristics of the benzene ring skeleton (C=C).^31^ The sharp peak at 1028 cm^−1^ is the stretching vibration of the C–O bond, and the peak at 836 cm^−1^ is the C–H vibration peak and O–H vibration of the benzene ring.^32^ By comparing the curves of TA and TR, it was found that the peak shapes of TR at 1453 cm^−1^ and 1028 cm^−1^ became significantly wider, indicating that phenol successfully connected to the aromatic ring of TA. In addition, the peak at 836 cm^−1^ disappeared, which might be due to the offset of phenol access. It can be seen from the TR curve that phenol has been successfully introduced into the tannin skeleton, increasing more phenolic hydroxyl groups and active sites. The peaks at 2959 cm^−1^ and 2930 cm^−1^ are ascribed to CH_2_ and CH_3_ stretching.^33^ The 1396 cm^−1^ corresponds to the stretching vibration of COO-.^34^ The SPI curve shows a broad absorption peak around 3435 cm^−1^, which belongs to the stretching vibrations of –OH and –NH. The characteristic peaks observed at 1659 cm^−1^, 1537 cm^−1^ and 1239 cm^−1^ correspond to the amide I (C=O), II (N–H) and III band (C–N and N–H).^35^ After adding TR, the absorption band at 3435 cm^−1^ became broader, indicating that the phenolic hydroxyl groups were involved in the interaction between SPI and TR. Furthermore, the vibration peak of –CH_2_ has shifted, the intensity of the amide I band peak increases, the vibration peak of the amide II band changes from 1537 cm^−1^ to 1528 cm^−1^, and the absorption band of COO- moves from 1396 cm^−1^ to 1389 cm^−1^. This indicates that TR–SPI forms cross-links through hydrogen bonds between the hydroxyl groups and the amino group of SPI, as well as covalent bonds between the oxidized quinone ring of TR and the free amino group of SPI.^36,37^

**Fig. 5:**
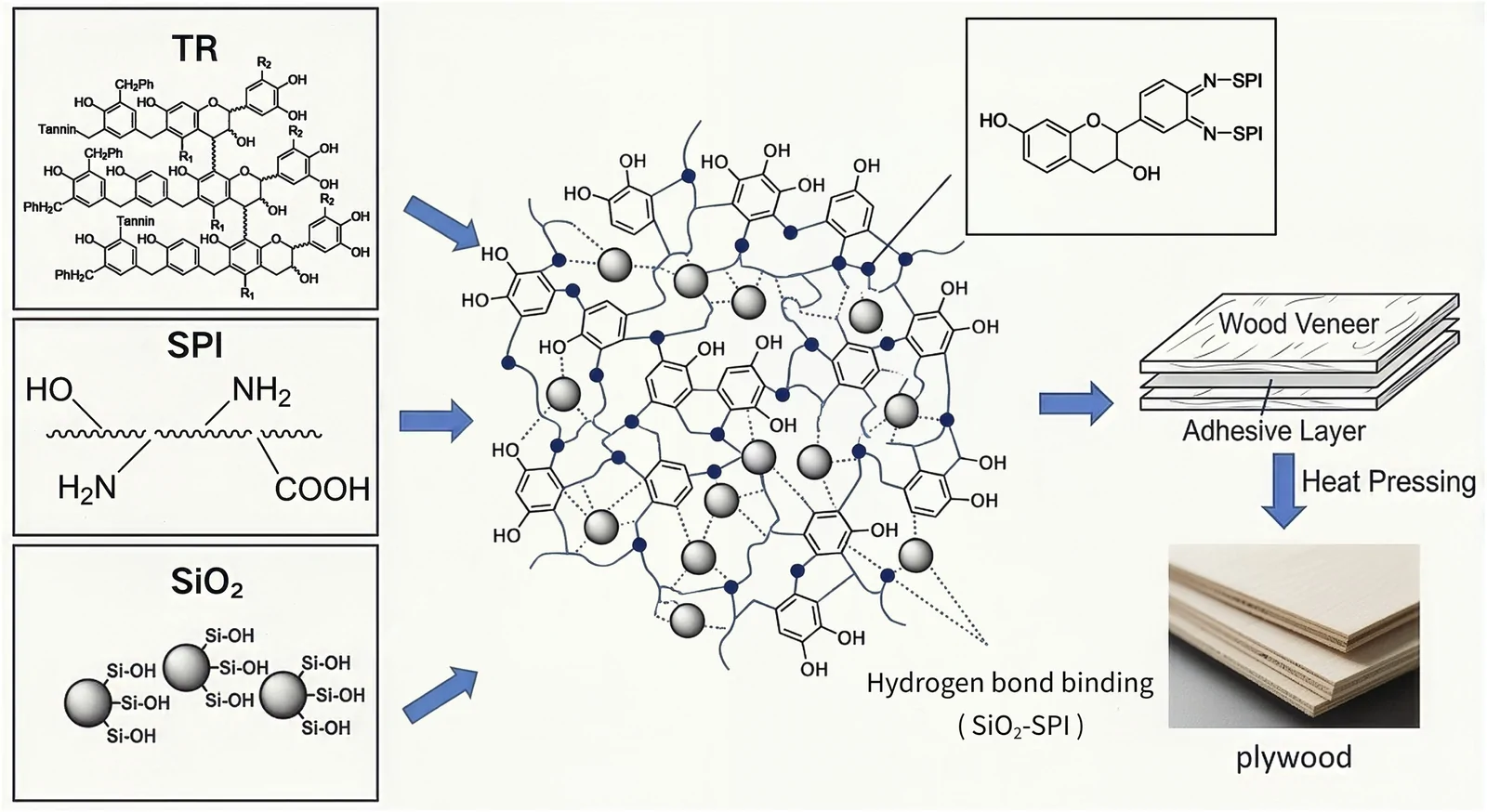
Tannin composite soy protein adhesive reaction mechanism.

After mixing TR resin and SPI adhesive, it can be seen from the curve that the absorption intensity of the characteristic peaks at 1608 cm^−1^, 1453 cm^−1^, and 1028 cm^−1^ has significantly weakened, indicating that the characteristic functional groups of the aromatic ring have chemically bonded with the amino and carboxyl groups of the SPI adhesive. In the SiO_2_–TR–SPI curve, in addition to retaining the characteristic absorption peaks of TR and SPI, a wide absorption band appears near 1100 cm^−1^. This is the asymmetric stretching vibration of the Si–O–Si bond, indicating that SiO_2_ has been successfully introduced into the system and may form hydrogen bonds with the active groups in the protein. This enhances the shear strength and water resistance of the adhesive, improves its mechanical properties, and is consistent with the test results in the experiment. The likely reaction mechanism was presented in Fig. 5.

### 3.4. Thermal properties analysis

**Fig. 6:**
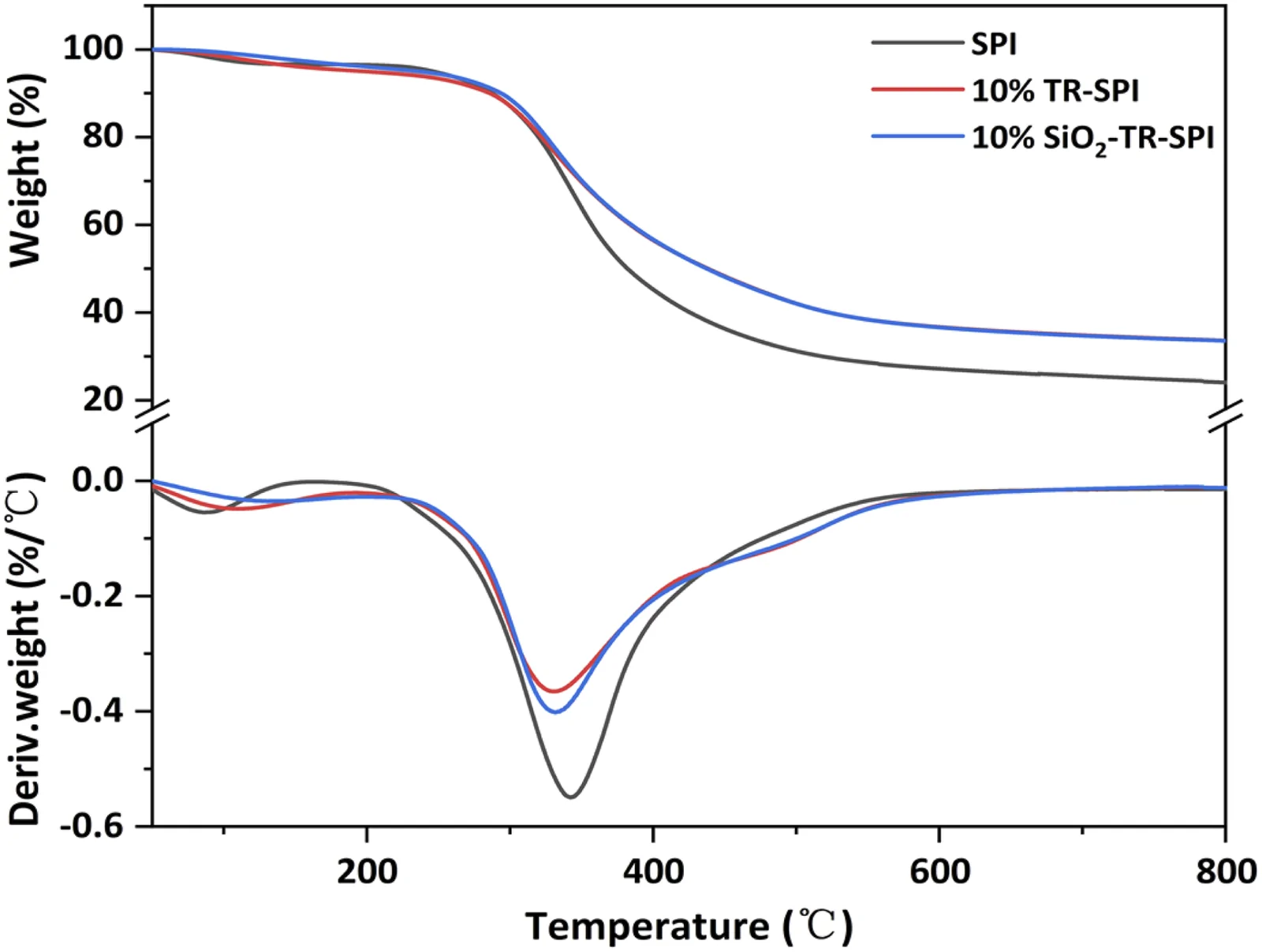
TG and DTG curves of SPI-series adhesives.

Fig. 6 shows the TG/DTG curve of the SPI series adhesive. Table 2 lists the maximum degradation rate temperature and residual mass rate of the adhesive. As shown in the figure, the thermal degradation process of all adhesives is divided into three stages. The first stage occurred at 50–200 °C, mainly the evaporation of residual moisture in the adhesive system. At this temperature, soy protein remains stable without decomposition, and the mass loss rate in this stage is usually very small.^38^ The second stage occurred at 200–400 °C, during which the main issue is the breaking of unstable chemical bonds in the adhesive system.^39^ Involving the breakage of hydrogen bonds and electrostatic bonds between molecules, as well as the breakage of covalent peptides that bind to the amino acid residues of SPI. It can be seen from the DTG curve that the SPI adhesive has the maximum degradation peak at 341.69 °C. This is because the intermolecular forces in the SPI system are mainly hydrogen bonds, and the crosslinking density is relatively low, thus the thermal stability is limited.^40^ After introducing TR into the SPI adhesive, the maximum degradation peak temperature decreased to 328.27 °C, indicating that the introduction of TR promoted the crosslinking reaction and increased the crosslinking density of the system. After further introducing SiO_2_ into the TR–SPI system, the maximum degradation rate was shown at 330.76 °C. Combined with FT-IR analysis, it can be known that monomer can chemically bond with amino and other groups of SPI to form a cross-linked network. The addition of SiO_2_, through the filling effect of its nanoparticles, jointly enhances the thermal stability of the adhesive. Moreover, this strong and continuous network structure also helps to improve the thermal resistance of the nanohybrid.^41,42^ The third stage occurred at 400–600 °C, mainly involving the degradation of the soy protein skeleton structure.^43^ This is related to the breakage of O–N, C–O and S–S bonds in SPI, as well as the breakage of main chain peptide bonds.^44^ According to the TG and DTG curves, the thermal stability performance of the SPI adhesive modified by adding TR and SiO_2_ was improved.

**Table 2:** Thermal properties of different adhesive samples

Samples	*T*_max_ a (°C)	*R*_w_ b (%)
SPI	341.69	24.12
10% TR–SPI	328.27	33.55
10% SiO_2_–TR–SPI	330.76	33.52

a Temperature of maximum rate of degradation.

b Residual mass rate.

## 4. Conclusions

In this study, using silica as the filler, a high-performance tannin-based resin composite soy protein adhesive was successfully prepared by synthesizing a tannin-based resin composite soy protein adhesive. When the addition amount of TR in the TR–SPI adhesive is 10%, the wet shear strength of the adhesive under the wet heat condition of 63 °C reaches 0.84 MPa, which has met the requirements of the national standard. The wet shear strength of the SiO_2_–TR–SPI adhesive prepared by further adding 5% SiO_2_ was increased to 1.47 MPa and 1.59 MPa respectively in the high-temperature and humid environment of 63 °C and 93 °C, far exceeding the national standard. The hot-pressing temperature experiment shows that there is no significant difference in bonding strength between 180 °C and 160 °C, confirming that the hot-pressing temperature of this system is moderate and it is easy to cross-link. Infrared spectroscopy analysis shows that the phenolic hydroxyl groups in TR undergo Schiff base reaction with the amino groups of soy protein, forming a cross-linked network. The SiO_2_–TR–SPI adhesive prepared by SiO_2_ through the dual effects of physical filling and chemical bonding has a denser cross-linked structure and stronger water resistance. This research provides a new idea for the development of high-performance and low-cost environmentally friendly adhesives, and is of great significance for promoting the high-value utilization of forestry resources.

## Author contributions

Xun Zhou: writing – original draft, writing – review and editing, conceptualization, investigation. Yunfeng Tao: writing – review and editing, methodology, formal analysis. Hualong Xuan: software, validation. Yixi Zhang: data curation, validation. Huali Tang: writing – review and editing, resources, supervision, funding acquisition.

## Conflicts of interest

There are no conflicts to declare.

## Data Availability

The data supporting the results of this study are included in this article, or can be obtained from the corresponding author upon reasonable request.
